# Evaluation of the Association between Menstrual Cycle Irregularity and Dental Pain or Chewing Discomfort in Women before Menopause

**DOI:** 10.3390/jcm8040454

**Published:** 2019-04-04

**Authors:** In-Seok Song, Eun Young Ki, Kyungdo Han, Jae-Jun Ryu, Jun-Beom Park

**Affiliations:** 1 Department of Oral and Maxillofacial Surgery, Korea University Anam Hospital, Seoul 02841, Korea; sis80@naver.com; 2 Department of Obstetrics and Gynecology, College of Medicine, The Catholic University of Korea, Seoul 06591, Korea; mdkey@catholic.ac.kr; 3 Department of Biostatistics, College of Medicine, The Catholic University of Korea, Seoul 06591, Korea; hkd917@naver.com; 4 Department of Prosthodontics, Korea University Anam Hospital, Seoul 02841, Korea; koprosth@gmail.com; 5 Department of Periodontics, College of Medicine, The Catholic University of Korea, Seoul 06591, Korea

**Keywords:** epidemiology, mastication, menstrual cycle, nutrition surveys, oral health, toothache

## Abstract

This study was performed to assess the relationship between menstrual irregularity and dental pain or chewing discomfort in women before menopause, using nationally representative data. This study analyzed 4595 participants who were ≥19 years or older, and did not have missing values for outcome variables from the Korean National Health and Nutrition Examination Survey. Tooth pain was considered present if the participant felt throbbing discomfort, pain, or sensitivity when eating hot or cold food or drinking hot or cold beverages. Self-reported oral chewing discomfort was obtained. Adjusted odds ratios and their 95% confidence intervals for tooth pain in the individuals with menstrual cycle irregularity were 1.30 (1.05, 1.62) after adjustment for age, body mass index, drinking, smoking, income, exercise, stress, metabolic syndrome, and the frequency of tooth brushing. Adjusted odds ratios and their 95% confidence intervals for chewing discomfort in the individuals with menstrual cycle irregularity were 1.33 (1.03, 1.72) after adjustment. The association between menstrual irregularity and dental pain or chewing discomfort in women before menopause was proven—after adjusting for confounding factors—by multiple logistic regression analyses. Menstrual cycle irregularity may be considered a potential risk indicator for dental pain or chewing discomfort in Korean women before menopause.

## 1. Introduction

Menstrual cycles can be considered important indicators of health and fertility [1,2]. Previous studies have shown the association between menstrual cycle irregularity and systemic diseases. Menstrual cycle length has been suggested as a predictor of cardiovascular disease and a breast cancer risk factor [3]. Women with long (a usual cycle length of 40 days or more) or highly irregular menstrual cycles have a significantly increased risk for developing type 2 diabetes mellitus [4], and the prevalence of type 2 diabetes is higher among women with a history of menstrual irregularity [5]. Moreover, it was reported that menstrual cycle irregularity can be considered a marker of metabolic disorders [6].

The effect of menstrual cycle on oral health has been suggested previously [2,7,8]. Fluctuation in estrogen/progesterone levels has been shown to affect the periodontium [7]. Sex hormones are reported to have the ability to proliferate specific periodontal microorganisms and affect host immunologic response [8]. Moreover, it has been shown that there is an association between menstrual cycle irregularities and periodontal treatment needs [2]. It was hypothesized that there is no significant association between menstrual irregularity and dental pain or chewing discomfort. Thus, this study was performed to assess the relationship between menstrual irregularity and dental pain or chewing discomfort in women before menopause using nationally representative data, and this is the first study to evaluate this topic.

## 2. Experimental Section

### 2.1. Participants

This study is a secondary data analysis of the Korean National Health and Nutrition Examination Survey (KNHANES) V, conducted in 2010–2012 by the Korean Ministry of Health and Welfare. The KNHANES, which is conducted annually to monitor the general health and nutritional status of the Republic of Korea population, is composed of a health interview survey, a health examination survey, and a nutritional survey by the trained staff members. A rolling sample design involving stratified, complex, and multistage probability samples was applied to gather the data. The Institutional Review Board of the Korea Centers for Disease Control approved this KNHANES and informed consent was obtained from all participants. Approval of this study was obtained by the Institutional Review Board at the Catholic University of Korea. This research was conducted based on the Helsinki Declaration-based ethical principles for medical research involving human subjects. All data analyzed during this study are included in this published article. The complete data for this study was obtained at [9].

### 2.2. Anthropometric Measurement and Definition of Variables

A total of 25,534 individuals were candidates in the KNHANES and we selected participants ≥19 years. Individuals who had missing values for outcome variables were excluded from this study. Finally, 4595 subjects were included in our analysis.

Trained staff members carried out anthropometric measurements. Participants wore light clothing when measuring body weight and height. Body mass index (BMI) was defined using the following formula: BMI = weight (kg)/height (m^2^). Waist circumference was measured at the approximate midpoint between the lower margin of the last palpable rib and top of the iliac crest at the end of a normal expiration.

Individuals were categorized using the criterion for alcohol consumed within one month based on the respondents’ responses on the self-reported questionnaire [10]. Smoking status was categorized as current smoker or not from the interview. Individuals were regarded as exercisers if they performed moderate exercise more than 5 times per week for over 30 min per session, or performed vigorous exercise more than 3 times per week for over 20 min per session [11]. Recognition of stress was self-reported. Data regarding menstrual cycle irregularity was collected by asking the participants to recall the duration of the menstrual cycle, and menstrual cycle characteristics were categorized as regular or irregular.

Blood samples were collected from the participants’ antecubital vein after fasting >8 h, and concentrations of serum fasting plasma glucose, total cholesterol, triglycerides, and high-density lipoprotein cholesterol were calculated. The American Heart Association/National Heart, Lung, and Blood Institute provided the Scientific Statement of the criteria for Asians for metabolic syndrome [12]. Three or more of the following criteria must be satisfied to be diagnosed with metabolic syndrome: elevated triglycerides (150 mg/dL or greater) or use of lipid-lowering medication; high-density lipoprotein cholesterol of lower than 40 mg/dL in men or lower than 50 mg/dL in women, or use of medication; waist circumference of 90 cm or higher in men or 80 cm or higher in women; blood pressure of 130/85 mm Hg or higher or use of antihypertensive medication; and fasting blood glucose of 100 mg/dL or higher or current use of antidiabetic medication.

### 2.3. Oral Health Behaviors and Definition of Number of Natural Teeth

Tooth pain was considered present if the participant felt throbbing discomfort, pain, or sensitivity when eating hot or cold food or drinking hot or cold beverages. Self-reported oral chewing discomfort was obtained. The time of day when participants brushed their teeth was recorded as oral health behavior [13]. We calculated the frequency of daily tooth brushing by the total number of times the teeth were brushed each day. The number of natural teeth was calculated when primary or permanent teeth were present.

### 2.4. Statistical Analysis

The results of this study are demonstrated as means ± standard errors for continuous variables, and as proportions (standard errors) for categorical variables. Logarithmic transformations were applied for variables having skewed distributions, when it seemed necessary. The differences in characteristics according to the presence of menstrual cycle irregularity were evaluated by a chi-square test for categorical variables or an independent *t*-test for continuous variables. A hierarchical multivariable logistic regression analysis was used to evaluate the risk of tooth pain and chewing discomfort in relation to menstrual cycle irregularity.

The risk of tooth pain and chewing discomfort was identified by evaluating the odds ratios and 95% confidence intervals. In model two, adjustments were made for age, body mass index, smoking, drinking, exercise, and income. Model three was adjusted like model 2 plus metabolic syndrome, stress, and the frequency of tooth brushing. SAS software version 9.2 was used for statistical analyses (SAS Institute, Cary, NC, USA). Statistical significance was set at *P*-value of <0.05.

## 3. Results

Table 1 describes the baseline characteristics of the study individuals according to the menstrual cycle irregularity. Body mass index, smoking, white blood cell count, and metabolic syndrome were significantly higher in participants with menstrual cycle irregularity. Higher education was significantly lower in participants with menstrual cycle irregularity.

**Figure 1:**
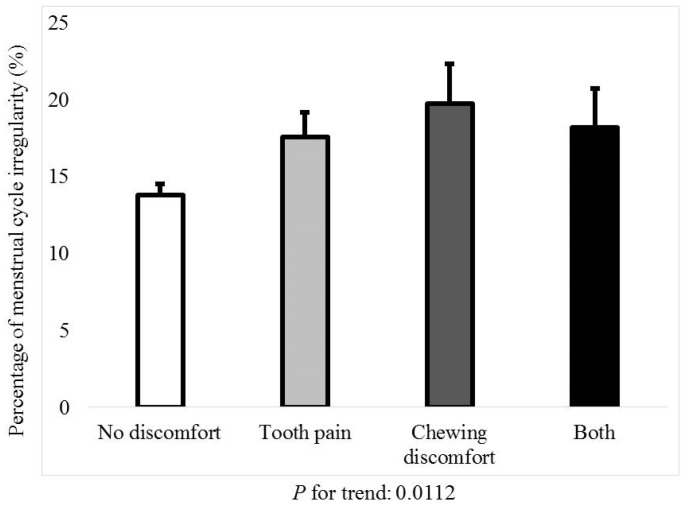
Percentage of menstrual cycle irregularity in the individuals with tooth pain and/or chewing discomfort.

**Figure 2:**
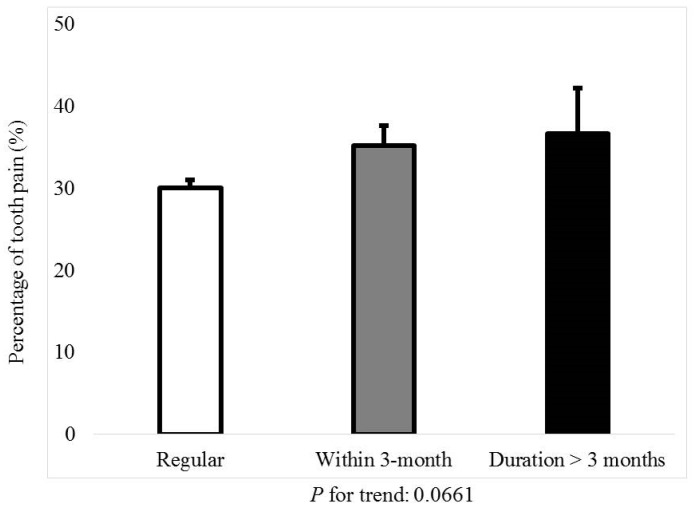
Percentage of tooth pain according to menstrual cycle categorization of regular, irregular (once within 3 months), and duration longer than 3 months.

**Figure 3:**
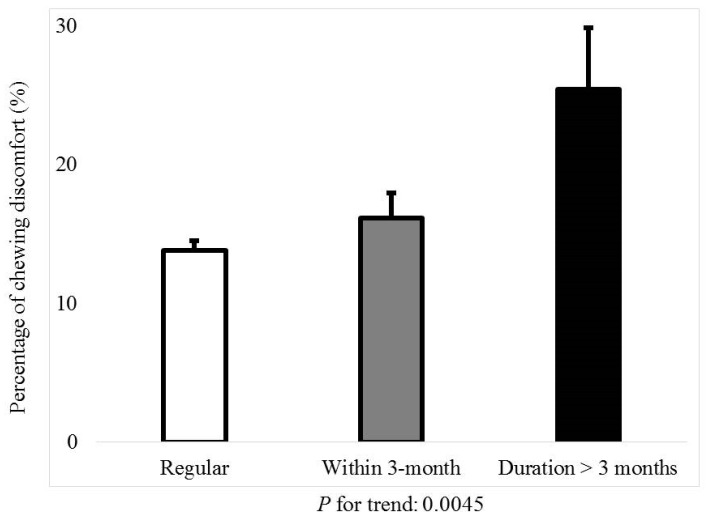
Percentage of chewing discomfort by menstrual cycle irregularity.

Figure 1 shows the percentage of menstrual cycle irregularity regarding the presence of tooth pain or chewing discomfort (*P* for trend: 0.0112). Figure 2 shows the percentage of tooth pain regarding the menstrual cycle; the *P*-value for the trend was 0.0661. The percentage of individuals with tooth pain was 30.0 ± 1.0% for regular menstrual cycles. The percentages of individuals with tooth pain with irregular menstrual cycles of once in three months and duration longer than three months were 35.1 ± 2.5% and 36.7 ± 5.5%, respectively. Figure 3 shows the percentage of chewing discomfort regarding the menstrual cycle (*P*-value for trend <0.01). The percentages of individuals with chewing discomfort were 13.8 ± 0.7%, 16.1 ± 1.8%, and 25.4 ± 4.4% for individuals with regular menstrual cycles, irregular menstrual cycles of once in three months, and duration longer than three months, respectively.

Table 2 shows the effects of oral health on the menstrual cycle irregularity. The percentage of tooth pain, chewing discomfort, and speech discomfort was statistically higher with menstrual cycle irregularity (*P* < 0.05). Table 3 shows the adjusted odds ratios and their 95% confidence intervals from multivariate logistic regression analyses for individuals with tooth pain and chewing discomfort. Adjusted odds ratios and their 95% confidence intervals for tooth pain in individuals with menstrual cycle irregularity were 1.30 (1.05, 1.62) after adjustment for age, body mass index, smoking, drinking, exercise, income, metabolic syndrome, stress, and frequency of tooth brushing. Adjusted odds ratios and their 95% confidence intervals for chewing discomfort in individuals with menstrual cycle irregularity were 1.33 (1.03, 1.72) after adjustment.

## 4. Discussion

This association may be explained by the following. Risk factors in the menstrual cycle were evaluated, and it was suggested that psychological stress and obesity may be related to menstrual cycle characteristics [14]. The results of this study showed that stress was significantly higher in participants with menstrual cycle irregularity. It was noted that cycle irregularity was associated with psychosocial stress [15]. High stress levels—greater than 20 using the Perceived Stress Scale—were associated with menstrual cycle irregularity [16]. High levels of psychological distress were significantly associated with poor self-rated oral health after adjustment for confounding factors [17]. Moreover, stress may alter the hypothalamic-pituitary-ovarian axis [18,19], and a shift in the axis may produce changes in pain sensitivity. One report showed that the menstrual cycle was associated with pain experience, and the increase in pain perception among females during their perimenstrual period was significantly greater than their postmenstrual period [20].

This study indicated that body mass index was higher for participants with menstrual cycle irregularity. Similarly, a previous report showed that menstrual cycle irregularity was associated with overall obesity [21]. Obese individuals had higher odds of having tooth loss [22]. The mean decayed, missing, and filled teeth index value was higher in obese participants when compared with nonobese individuals [23], and a significant positive correlation between body mass index and dental caries was suggested after controlling for potential confounders [24]. Similarly, body mass index has been shown to exert an influence on masticatory performance [25]. In obese women, especially with central obesity, the absorbed steroid is transformed into estrogen in peripheral fat [26,27], and the change in the level of estrogen may produce tooth pain [28]. However, another study reported that body mass index was related to periodontitis but not to dental caries [29]. In another report, dental caries had no significant relationship with abdominal obesity [30]. Menstrual cycle irregularity was suggested to be a potential risk indicator for periodontal disease [2], which may explain the association between menstrual cycle irregularity and chewing discomfort.

Disordered eating is strongly related to menstrual irregularity [31]. Menstrual regularity can be influenced by specific dietary nutrients that may have direct effects or exert their effects by modulating circulating sex steroid status [32]. The incidence of menstrual irregularity was 4.9% among non-vegetarians and 26.5% among vegetarians [32]. Thus, diet may explain the relationship between menstrual cycle irregularity and dental pain.

Various methods have been used to assess menstrual cycle characteristics [2,33,34]. Our previous report categorized the menstrual cycle as regular, irregular (once within three months), or duration longer than three months [2]. In another report, the periods were defined as regular if the overall range was within 20 to 40 days [33]. In other research, menstrual cycle difference was calculated as the difference (in days) between the longest and shortest menstrual cycle in the past 12 months, and the menstrual cycle was considered irregular if the difference was ≥15 days [33,34]. These various methods for evaluating menstrual irregularity in each research may produce different results.

This study had several limitations. Firstly, the measurement of menstrual regularity is at best an approximation of female reproductive status, because regular cycles may, in fact, be anovulatory or mask luteal-phase inadequacies that can be detected only by direct hormone-concentration measurements [32]. Secondly, the exact length of the cycle was not reported, and issues may arise from the use of recalled menstrual histories [32]. A previous study reported that there were considerable measurement errors in self-reported cycle length and the recollection of menstrual cycle length [35,36]. However, the collection of information regarding menstrual cycle by survey is a widely used method [37]. KNHANES applied a rolling sampling design while obtaining the data used in this study, which involves complex, stratified, and multistage probability samples—and these methods allows the data to be considered as a nationally representative result. The association between menstrual irregularity and dental pain or chewing discomfort in women before menopause was discovered by multiple logistic regression analyses after adjusting for confounding factors, and consequently, the results can be considered representative and reliable.

## 5. Conclusions

The association between menstrual irregularity and dental pain or chewing discomfort in women before menopause was proven—after adjusting for confounding factors—by multiple logistic regression analyses. Menstrual cycle irregularity may be considered a potential risk indicator for dental pain or chewing discomfort in Korean women before menopause.

## Figures and Tables

**Table 1:** Baseline characteristics of study participants according to menstrual cycle irregularity.

	Menstrual Cycle Irregularity		
	No	Yes	*P*-Value *
Unweighted n	3940	655	
Tooth pain (yes)	30.0 (1.0)	35.4 (2.3)	0.02
Chewing (discomfort)	13.8 (0.7)	17.37 (1.7)	0.02
Age (years)	35.5 ± 0.2	34.9 ± 0.5	0.27
Body mass index (kg/m^2^)	22.4 ± 0.1	23.2 ± 0.2	<0.01
Alcohol within one month	52.3 (1)	48.8 (2.5)	0.18
Smoking (currently)	5.9 (0.5)	8.8 (1.4)	0.02
Exercise (yes)	16.9 (0.8)	17.5 (1.8)	0.77
Income (the lowest quartile)	8.5 (0.7)	9.2 (1.5)	0.63
Education (high school graduate or higher)	44.9 (1.1)	31.5 (2.3)	<0.01
White blood cell (×10^9^/L) **	74.6 ± 0.2	76.4 ± 0.5	<0.01
Stress (yes)	32.3(0.9)	39.8 (2.2)	<0.01
Metabolic syndrome	10.4 (0.6)	14.3 (1.6)	0.01
Number of natural teeth	27.1 ± 0.03	26.92 ± 0.11	0.13
Frequency of tooth brushing per day			<0.01
≤1	3.8 (0.4)	5.8 (1.0)	
2	37.6 (1.0)	42.7 (2.3)	
≥3	58.5 (1.0)	51.6 (2.5)	
Speech (discomfort)	2.7 (0.3)	5.5 (1.1)	<0.01
Dental checkup within 1 year (yes)	12.7 (0.7)	9.4 (1.3)	0.04

Data are presented as means ± standard error or percentages (standard error). * *P*-values were obtained by independent *t*-test for continuous variables or chi-square test for categorical variables. ** Log transformation was applied to the value, and geometric mean (95% confidence interval) is shown.

**Table 2:** The effects of oral health on menstrual cycle irregularity.

	Menstrual Cycle Irregularity		
	No	Yes	*P*-Value
n	3940	655	
Tooth pain (yes)	30.0 (1.0)	35.4 (2.3)	0.02
Chewing (discomfort)	13.8 (0.7)	17.7 (1.7)	0.02
Frequency of tooth brushing per day			0.01
≤1	3.8 (0.4)	5.8 (1.0)	
2	37.6 (1)	42.7 (2.3)	
≥3	58.5 (1)	51.6 (2.5)	
Speech (discomfort)	2.7 (0.3)	5.5 (1.1)	<0.01
Dental checkup within 1 year (yes)	12.7 (0.7)	9.4 (1.3)	0.04

Data are presented as percentages (SE).

**Table 3:** Adjusted odds ratios and 95% confidence intervals of the individuals with tooth pain or chewing discomfort in multivariate logistic regression models in the presence of menstrual cycle irregularity.

	Tooth Pain		Chewing Discomfort	
	Odds Ratio (95% Confidence Interval)	*P*-Value	Odds Ratio (95% Confidence Interval)	*P*-Value
Model 1	1.23 (1.05, 1.60)	0.02	1.35 (1.05, 1.74)	0.02
Model 2	1.28 (1.04, 1.58)	0.02	1.37 (1.06, 1.77)	0.02
Model 3	1.30 (1.05, 1.62)	0.02	1.33 (1.03, 1.72)	0.03

Model 1: No adjustment. Model 2: Model 1 + age, body mass index, smoking, drinking, exercise, and income adjusted. Model 3: Model 2 + metabolic syndrome, stress, and the frequency of tooth brushing adjusted.
